# Small-diameter hybrid vascular grafts composed of polycaprolactone and polydioxanone fibers

**DOI:** 10.1038/s41598-017-03851-1

**Published:** 2017-06-15

**Authors:** Yiwa Pan, Xin Zhou, Yongzhen Wei, Qiuying Zhang, Ting Wang, Meifeng Zhu, Wen Li, Rui Huang, Ruming Liu, Jingrui Chen, Guanwei Fan, Kai Wang, Deling Kong, Qiang Zhao

**Affiliations:** 1 State Key Laboratory of Medicinal Chemical Biology, Key Laboratory of Bioactive Materials, Ministry of Education, College of Life Sciences, Nankai University, Tianjin, 300071 China; 2 Urban Transport Emission Control Research Centre, College of Environmental Science and Engineering, Nankai University, Tianjin, 300071 China; 3 Center for Research and Development of Chinese Medicine, Tianjin State Key Laboratory of Modern Chinese Medicine, Tianjin University of Traditional Chinese Medicine, Tianjin, 300193 China

## Abstract

Electrospun polycaprolactone (PCL) vascular grafts showed good mechanical properties and patency. However, the slow degradation of PCL limited vascular regeneration in the graft. Polydioxanone (PDS) is a biodegradable polymer with high mechanical strength and moderate degradation rate *in vivo*. In this study, a small-diameter hybrid vascular graft was prepared by co-electrospinning PCL and PDS fibers. The incorporation of PDS improves mechanical properties, hydrophilicity of the hybrid grafts compared to PCL grafts. The *in vitro/vivo* degradation assay showed that PDS fibers completely degraded within 12 weeks, which resulted in the increased pore size of PCL/PDS grafts. The healing characteristics of the hybrid grafts were evaluated by implantation in rat abdominal aorta replacement model for 1 and 3 months. Color Doppler ultrasound demonstrated PCL/PDS grafts had good patency, and did not show aneurysmal dilatation. Immunofluorescence staining showed the coverage of endothelial cells (ECs) was significantly enhanced in PCL/PDS grafts due to the improved surface hydrophilicity. The degradation of PDS fibers provided extra space, which facilitated vascular smooth muscle regeneration within PCL/PDS grafts. These results suggest that the hybrid PCL/PDS graft may be a promising candidate for the small-diameter vascular grafts.

## Introduction

Cardiovascular disease (CVD) is the NO.1 killer in the world, and it is responsible for >17.3 million deaths every year, a number that represents 31% of all global deaths^1^. Bypass surgery has been one of the most effective treatments for CVD, and helped patients live longer. The artificial blood vessel have shown great potential applications in bypass surgery, and provided a good alternative to the autologous vein. Although three kinds of commercialized vascular grafts, expanded polytetrafluoroethylene (ePTFE), poly(ethylene terephthalate) (Dacron) and polyurethane (PU), have been used in vascular surgery almost 50 years as large diameter prostheses, intimal hyperplasia and thrombosis limit the application of these polymer in smaller diameter grafts (<6mm)^2^. Thus, it is very important to find an appropriate biodegradable material for fabrication of smaller diameter grafts.

PCL is a Food and Drug Administration (FDA)-approved biodegradable polymer with wide applications in tissue engineering^3^. Many studies have shown that electrospun PCL vascular grafts have remarkable patency because of its optimal hemocompatibility and mechanical properties^4–7^. But the number of cells and capillaries in the electrospun PCL vascular graft walls decreased on the long term (12 and 18 months), meanwhile, calcification was observed, due to the slow degradation, lower compliance and dense fibrous structure^4^. Our group fabricated an electrospun PCL vascular graft with thick fibers and large pores in order to enhance cell infiltration, vascularization and efficient regeneration of functional tunica media^6^. Although the grafts have much better effects in vascular regeneration and remodeling compared to thin fiber PCL grafts, the slow degradation of PCL is still a problem.

A fast-degrading vascular graft was made of elastic poly (glycerol sebacate) (PGS), and wrapped with a dense electrospun PCL sheath which enhanced graft strength and prevented bleeding^8^. The graft regenerated into compliant neoarteries resembled native arteries in the components and structures of tissue within 3 months. Rapid degradation can reduce inflammatory responses caused by long-lasting materials, in addition, and also provide more space for cell infiltration and extracellular matrix (ECM) secretion. However, the safety issue should be taken into account when the PGS grafts are used in large animals or human due to the rapid degradation of PGS which does not match the slow tissue regeneration process in large animals or human. To solve this problem, researchers combined fast-degrading and slow-degrading materials to fabricate vascular grafts with an appropriate degradation rate.

Baker *et al*. developed a dual-polymer composite scaffold containing PCL (a slow-degrading polyester) and poly(ethylene-oxide) (PEO) (a water-soluble polymer) via co-electrospun from two separate spinnerets^9^. The fast solution of PEO provided space for cell infiltration. But the excessive solution of PEO resulted in an overall loss in structural integrity of scaffold. In order to maintain scaffold integrity with an appropriate degradation rate, this group fabricated a multicomponent nanofibrous scaffold by a tri-jet electrospinning device. These fibers included PEO, PCL, and poly(lactide-co-glycolide) (PLGA) (medium-degrading) with different degradable rates^10^. After the PEO fibers fast dissolved, the PCL and PLGA fibers would serve to maintain scaffold integrity initially, and PLGA fibers gradually degraded to augment pore size and porosity. The presence of the three types of fibers provided time-dependent characteristics in the composite scaffold, and could also be used to further refine the mechanical properties.

PDS has been widely used as absorbable wound closure suture in clinical practice under the trade name PDS^®^, and approved by FDA^11^. PDS lost about 50% of original strength at four weeks *in vivo*, and completely absorbed after 240 days without obvious tissue reaction^12^. The degradation products of PDS had been proven low-toxicity, and caused a lower inflammatory response rates than Vicryl (PLGA) and Dexon (poly(glycolic acid))^13^. Compared to polyglycolide (PGA) and Dacron synthetic grafts, the grafts made of PDS have been shown to be less thrombogenic^14^. However, when tested *in situ* in a rat model alone, the insufficient cellular infiltration and fast degradation of PDO grafts led to aneurismal formations^15^.

Inspired from above previous reports, we fabricated a hybrid grafts consisting of fast-degrading PDS fibers and slow-degrading PCL fibers by co-electrospinning. We anticipated that fast degradation of PDS fibers can enlarge the pore spaces to promote cell infiltration and tissue regeneration, meanwhile, the slow-degrading PCL fibers maintained the structural integrity of scaffold and provided enough mechanical strength to prevent aneurysm. Here we describe the degradation behavior of the hybrid PCL/PDS scaffolds *in vivo*/*vitro* and tissue regeneration and remolding of the hybrid PCL/PDS grafts in rat abdominal aorta model.

## Results and Discussion

### The characterization of electrospun PCL/PDS grafts

**Figure 1:**
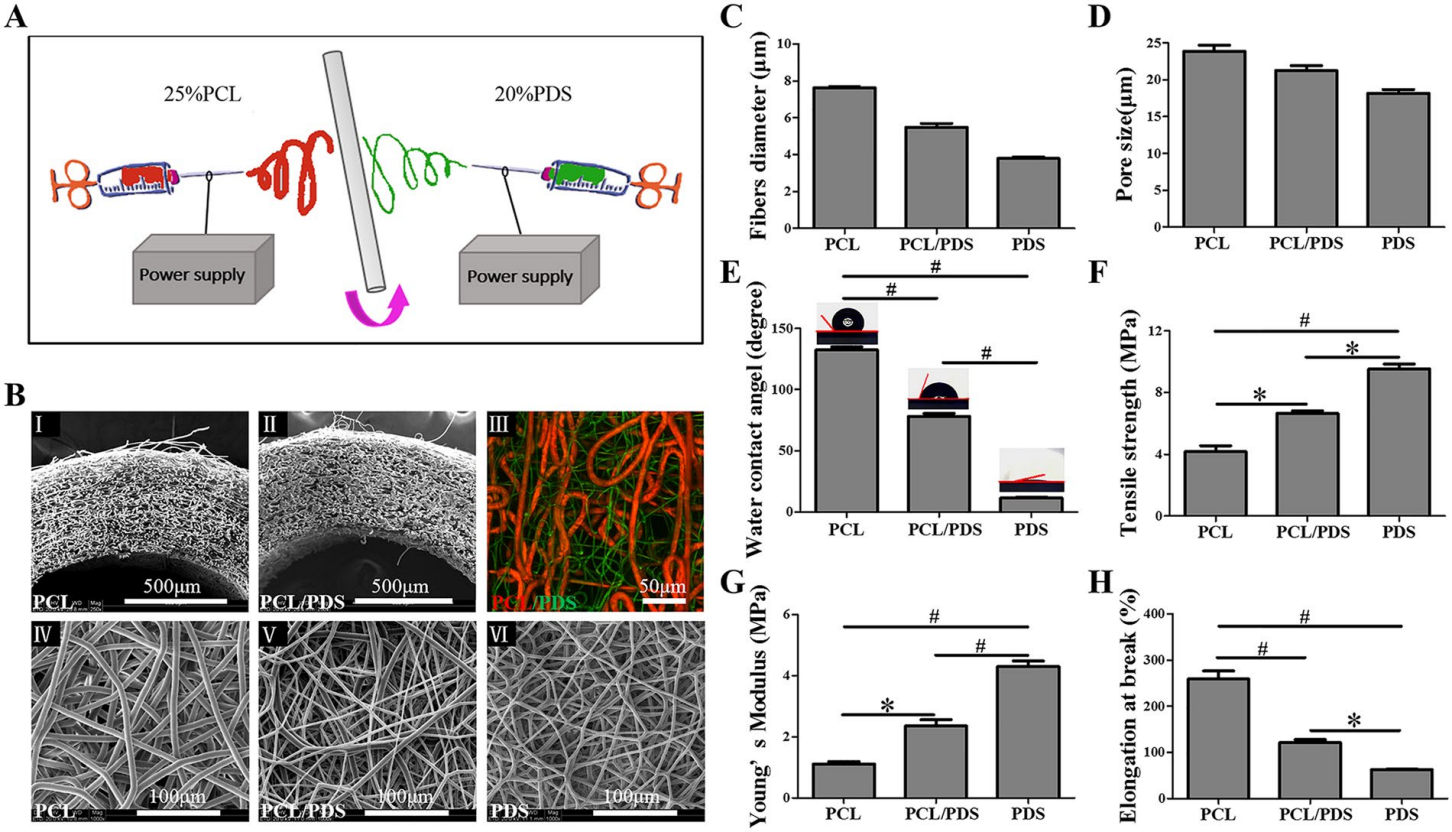
Characterization of vascular grafts. (**A**) The schematic diagram for preparation of hybrid PCL/PDS grafts. (**B**) The morphology of vascular grafts was evaluated by SEM and CLSM. SEM images of cross-section of PCL (I) and PCL/PDS grafts (II) showed that they had favorable tubular structure. Fluorescent images of hybrid PCL/PDS grafts with red PCL fibers labled with DiI and green PDS fibers labled with DiO (III). SEM images of lumen surface of vascular grafts (IV,V,VI) showed all the fibers had a smooth surface with well-defined fiber morphology. The average fibers diameter (**C**) and pore size (**D**) of vascular grafts was calculated based on the SEM images of grafts lumen surface. (**E**) Surface hydrophilic/hydrophobic performance analyzed by WCA analysis (n = 4), and the corresponding images of water droplets on the different surfaces after contact of 20 seconds. (**F**,**G** and **H**) The transverse mechanical properties of vascular grafts (n = 3). *p < 0.05, ^#^ *p* < 0.001.

Co-electrospinning has been widely used to fabricate vascular grafts due to its unique capacity for integrating the advantages of different biomaterials into a graft^16, 17^. Figure 1A is the schematic diagram for preparing hybrid grafts containing PCL and PDS fibers by co-electrospinning. Of the two fiber components, PCL fibers with slow degradation provided mechanical support and maintained tubular structural integrity, while the fast degradation of PDS fibers could provide extra space to facilitate vascular regeneration. The processing parameters of PCL fiber have been optimized with the aim of obtaining macroporous structure to facilitate tissue regeneration, which has been reported before^6^. In this study, the optimized PCL fibers were selected to prepared PCL/PDS vascular grafts. The relative ratio of two components is a key factor for this study. So we planned to fabricate a series of vascular grafts over a wide range of ratios (the ratio of PCL:PDS = 2.50:1; 1.67:1; 1:1; 0.6:1) by adjusting electrospun PDS parameters (Table S1). In terms of PDS fiber, the processing window is also very narrow with optimal flow rates of 6 mL/h. Further increasing the flow rate leads to the liquid leaking, while decreasing fails to obtain stable jet during the electrospinning. Therefore, only PCL/PDS vascular grafts with a theoretical ratio of 1.67 can be prepared and used for the following experiments.

SEM images showed PCL and PCL/PDS grafts exhibited a favorable tubular structure (Fig. 1B(I,II)). All spun fibers of the three types grafts had smooth surfaces with a well-defined fiber morphology, and no beads or defects were observed (Fig. 1B(IV–VI)). The fibers of large diameter were attributed to PCL, while those of small diameter correspond to PDS, by comparing with the SEM images of pure PCL and PDS grafts. In order to better discriminate the fibers distribution, two types of fiber was fluorescently stained, respectively. As shown in Fig. 1B(III), red PCL fibers labled with DiI and green PDS fibers labled with DiO were distributed randomly and uniformly. The ratio of PCL:PDS was 1.86 ± 0.14:1 (the weight percentage of PDS within PCL/PDS graft was 35.13 ± 2.87 wt%), which agreed well with the theoretical value determined according to the feeding ratio (the theoretical value of PCL:PDS was 1.67:1). After completely leaching PCL, the hybrid graft still maintained a good tubular structure (Supplementary Fig. S1), which also indicated that the distribution of the two kinds of fibers was randomly and uniformly.

The fiber diameter (Fig. 1C) and pore size (Fig. 1D) of grafts were also determined based on the SEM images. Although the average pore size of PCL/PDS grafts (20.06 ± 1.01 μm) were moderately decreased due to the addition of thinner PDS fibers, it was still large engough allowing the cellular infiltration (10 μm)^18^.

PCL is a hydrophobic polymer with water contact angle (WCA) of 131.28 ± 0.65° for electrospun PCL. After the incorporation of hydrophilic PDS component (11.54 ± 0.60°), the hydrophicity was improved, that is, the mean WCA of PCL/PDS decreased to 78.06 ± 2.20° (Fig. 1E). This was in agreement with our previous study that co-electrospun PCL and hydrophilic gelatin increased the hydrophicity of hybrid scaffolds^19^. Cell is usually apt to adhere on surfaces with a water contact angles of approximately 70°^20^. Compared to pure PCL and PDS grafts, the cells may be much easier to adhere on PCL/PDS grafts.

Many previous studies have indicated that electrospun PCL grafts had excellent mechanical properties^6, 21^. Compared to PCL grafts, the PCL/PDS grafts had a significant increase in Tensile strength and Young’s modulus (Fig. 1F,G). On the other hand, the Ultimate elongation at break of PCL/PDS grafts (121.31 ± 6.36%) was significantly decreased compared to that of PCL, whereas it was still higher than that of the native arteries (65–83%) (Fig. 1H)^22^. Therefore, the mechanical properties of PCL/PDS grafts could meet the requirement for vascular transplantation.

### Patency

**Figure 2:**
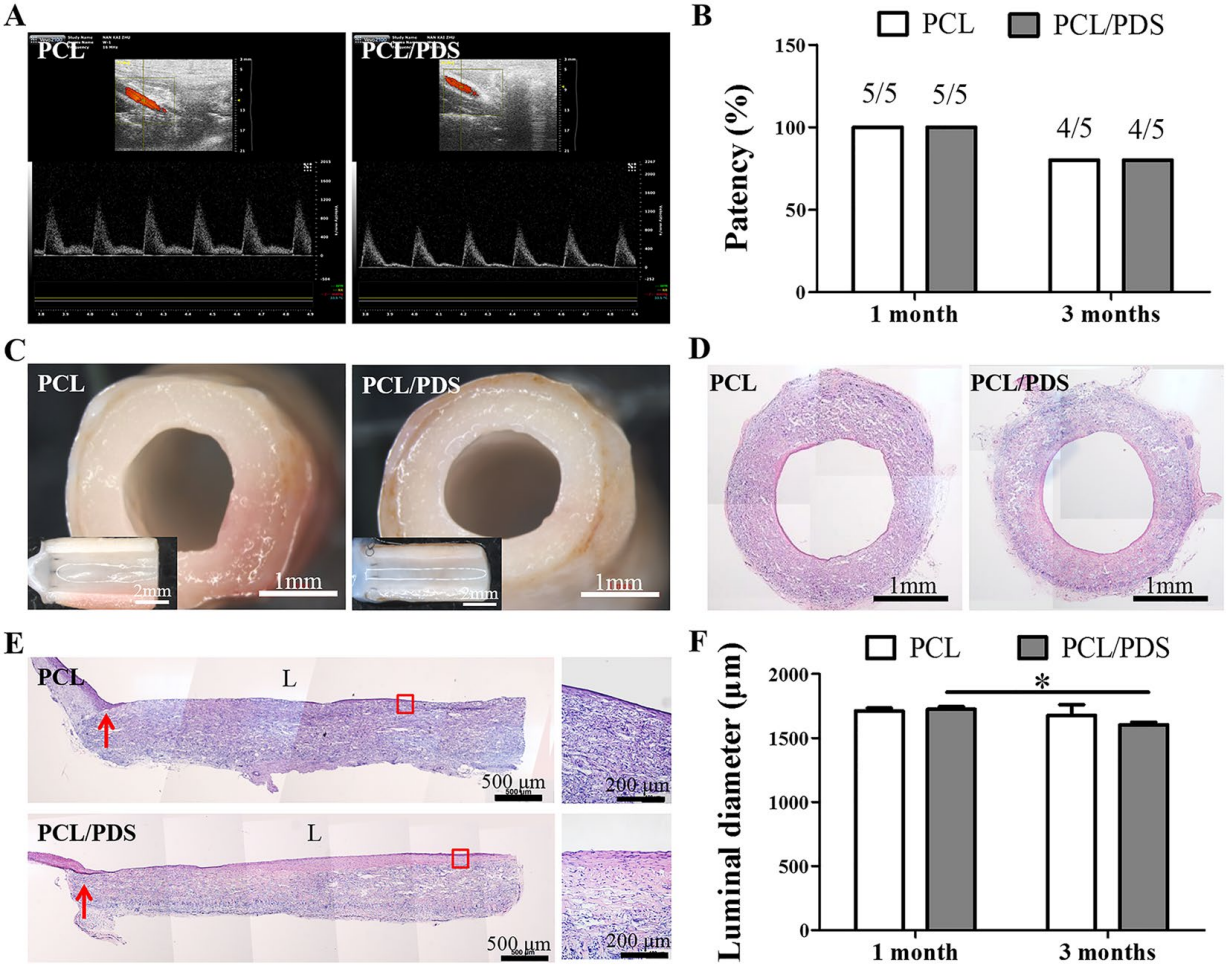
Evaluation of the patency and luminal diameter of explanted grafts at 3 months after implantation. (**A**) The patency was measured by color Doppler ultrasound. (**B**) The patency rates of the PCL and PCL/PDS grafts at both time points. (**C**) The lumen of the explanted grafts was smooth and free of thrombus under stereomicroscope. (**D**) Cross sections were stained with H&E to identify the neointima formation. (**E**) Representative H&E staining of longitudinal sections of explanted grafts. (**F**) The luminal diameter of explanted grafts was calculated based on the cross sections with H&E staining. (**L**) lumen; Red arrows: suture site. *p < 0.05.

Before implantation, the blood compatibility of PCL/PDS and PDS grafts was evaluated and further compared with that of PCL grafts to guarantee the security of implantation. The results of hemolysis, blood coagulation and fibrinogen absorption assays showed that there was no detectable difference among the three groups (Supplementary Fig. S2), indicating that the PCL/PDS hybrid grafts had good blood compatibility, similar to PCL graft. Subsequently, PCL/PDS and PCL grafts were implanted into rat abdominal aorta to replace a segment of native aorta. The color Doppler ultrasound showed that both grafts were patent at 1 month, while one occlusion occurred in both two groups at 3 months (Fig. 2A,B), which was due to over-proliferation of SMCs in native aorta stimulated by surgery. Compared with PCL grafts, the blood velocity of PCL/PDS grafts was closer to that of native abdominal aorta (Table 1).

**Table 1:** Blood velocity the grafts measured by color Doppler ultrasound.

Velocity	1 month		3 months		Abdominal aorta
	PCL	PCL/PDS	PCL	PCL/PDS	
Proximal velocity (mm/s)	1081.9 ± 80.9	1009.0 ± 59.5	705.80 ± 97.2	705.9 ± 52.4	
Graft velocity (mm/s)	959.2 ± 79.0	894.4 ± 54.2	577.7 ± 52.5	610.1 ± 46.1	846.6 ± 39.1
Distal velocity (mm/s)	1078.8 ± 53.6	1007.6 ± 68.4	577.7 ± 52.5	576.8 ± 36.7	

The degradation test *in vivo* and *in vitro* showed that the PDS fibers within PCL/PDS and PDS scaffolds begun to degrade at 2 weeks, and almost completely degraded at 12 weeks (Supplementary Fig. S3; Supplementary Fig. S4). Due to the fast degradation, the PDS grafts showed obvious aneurysmal dilatation at 1 month, therefore no further analysis and implantations were made (Supplementary Fig. S5). However, there was no aneurysmal dilatation and macroscopic thrombus occurred in any patent PCL and PCL/PDS grafts over the implantation period, which indicated that PCL provided necessary mechanical support for hybrid PCL/PDS grafts (Fig. 2C; Supplementary Fig. S6A). Based on the above results, we believed that mechanical property was the key factor in vascular grafts implantation. Thus we investigated the mechanical performance of PCL/PDS grafts after implantation. At 1 month, the mechanical properties (Tensile strength and Young’s modulus) of PCL/PDS grafts significantly decreased in comparison with as-spun grafts. The mechanical properties of PCL/PDS grafts were further lowered with increasing implantation time, and they were very close to native abdominal aorta without significant difference after 3 months (Supplementary Fig. S9).

Longitudinal sections were stained with H&E to identify the neointimal formation (Fig. 2E; Supplementary Fig. S6B). One month after implantation, the lumen surface of PCL/PDS grafts was almost covered by the neointima, but the mid-portion of PCL grafts showed some thrombin and incomplete neointimal coverage (Supplementary Fig. S6B). After 3 months, all grafts had been covered by neointima in both groups (Fig. 2E). Luminal diameter was determined based on H&E images (Fig. 2D; Supplementary Fig. S6C). From 1 to 3 months, luminal diameters were decreased overtime for both PCL and PCL/PDS grafts, but no stenosis were found. The narrowing was more significant in the PCL/PDS grafts (from 1723.83 ± 22.06 μm at 1 month to 1601.95 ± 21.77 μm at 3 months) than that in the PCL grafts (1713.07 ± 20.41 μm at 1 month to 1677.11 ± 84.11 μm at 3 months) (Fig. 2F). We also observed that similar phenomenon in the case of subcutaneous implantation experiment (Supplementary Fig. S4A,B). This phenomenon was ascribed to the bulky material property of the PDS. The different magnified H&E images of cross sections showed that the neointima of PCL/PDS grafts was closer to the SMCs layer of the native abdominal aorta and the two types of grafts had completed cellularization (Fig. S7). To evaluate the effect of PDS fibers degradation on cell migration, the cross-sections of explanted grafts were stained with DAPI, and cell density of graft walls were further quantified (Fig. S8). Statistical analysis confirmed that the cell density is similar between the PCL and PCL/PDS grafts at 1 month, while the cell density of PCL/PDS grafts (545.47 ± 22.58) is higher than that of PCL grafts (499.07 ± 18.86) at 3 months (Fig. S8B, *p* = 0.0523), which indicated that the larger pores generated by PDS degradation can enhance cells migration to some extent.

### Endothelialization

The lumen of native blood vessels is lined with a layer of ECs, the endothelium, which provides an anti-coagulating layer to prevent thrombosis^23^. Therefore, rapid endothelialization is a prerequisite for artificial vascular grafts.

**Figure 3:**
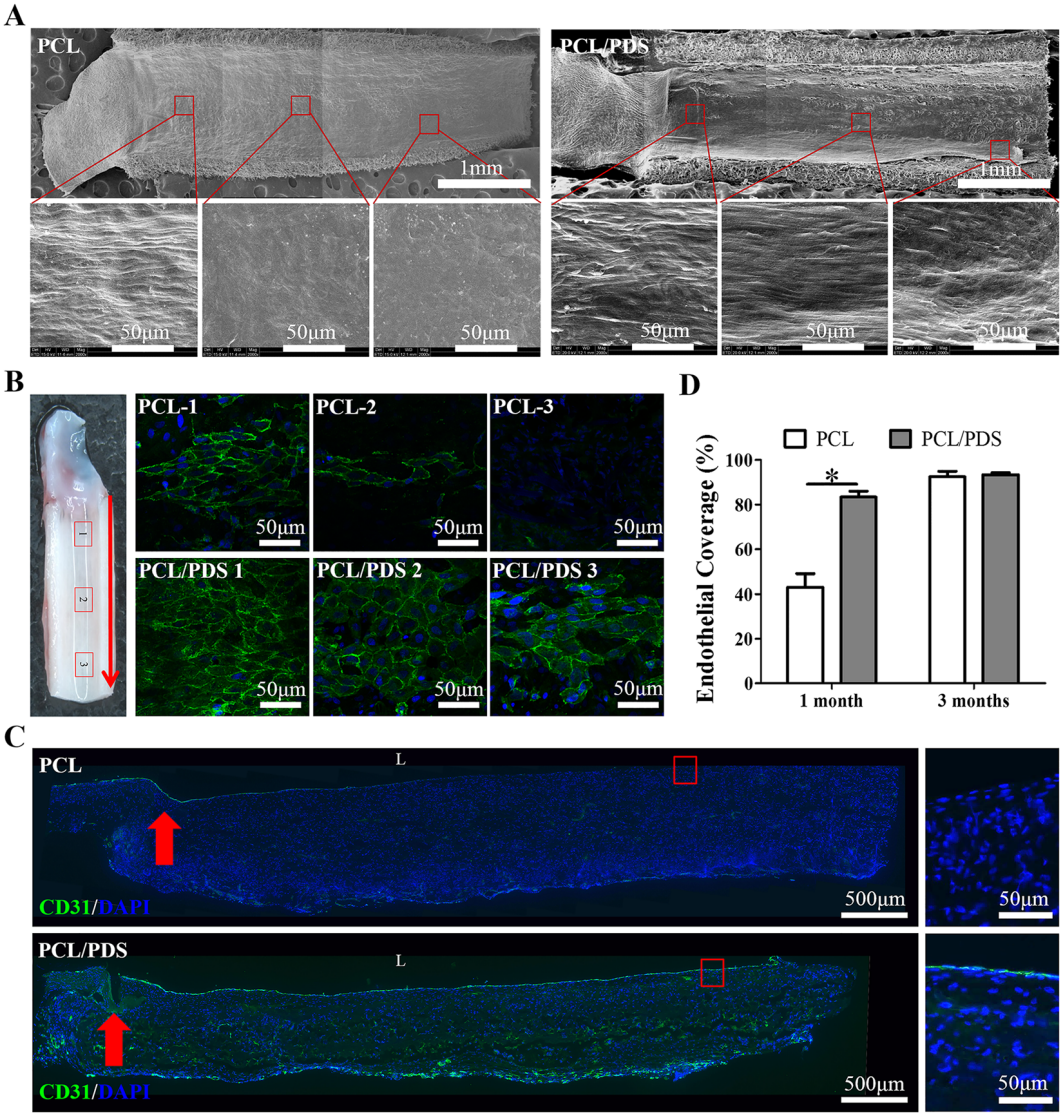
Endothelialization formation of the explanted grafts at 1 month after implantation. (**A**) The lumen surface of explanted grafts was observed by SEM. (**B**) The endothelial coverage of grafts was observed by *En face* immunostaining using CD31 antibody (Red arrows: blood flow direction). (**C**) Endothelialization was analyzed by immunofluorescence staining of longitudinal sections of grafts using CD31 antibody (L: lumen; Red arrows: suture site). (**D**) The endothelial coverage at 1 and 3 months was calculated based on the longitudinal sections with CD31 staining. *p < 0.05.

The lumen of the explanted grafts was observed by SEM to analyze the endothelium coverage at three different positions, the near suture site, the midportion and the site between them (Fig. 3A). At 1 month, the suture sites of all grafts were covered by ECs with cobblestone-like morphology, but the endothelial coverage was better in PCL/PDS grafts than in the PCL grafts in the other two positions. After 3 months, the luminal surfaces of all grafts were fully covered by ECs, but high magnified images showed that there was more collagen in the PCL/PDS graft wall (Supplementary Fig. S10A), which was similar to native abdominal aorta (Supplementary Fig. S10D).

*En face* staining of the lumen surface of grafts showed the coverage of CD31^+^ cells on the PCL/PDS grafts was faster than that on PCL grafts at 1 month (Fig. 3B), and the two groups almost completed host endothelialization at 3 months. More importantly, the CD31^+^ cells on PCL/PDS grafts showed more cobblestone-like morphology and alignment parallel to the direction of blood flow compared to PCL grafts (Supplementary Fig. S10B).

The endothelialization rates were analyzed based on the longitudinal sections with anti-CD31 antibody staining (Fig. 3C; Supplementary Fig. S10C). The ratio of endothelial coverage of PCL/PDS grafts (83.33 ± 2.66%) was significantly (*p* < 0.05) higher than that of the PCL grafts (43.02 ± 6.06%) at 1 month (Fig. 3D). The ratio of endothelial coverage of the two groups was almost close to 100% at 3 months. These results indicated that the PCL/PDS grafts could effectively enhance endothelium formation, especially at early time-point.

The rapid endothelium formation may be due to the hydrophilicity of PCL/PDS grafts (Fig. 1E). Compared to hydrophobic surface, hydrophilic surface is favorable to the adhesion, proliferation, and migration of ECs^24^.

### Vascular smooth muscle regeneration

**Figure 4:**
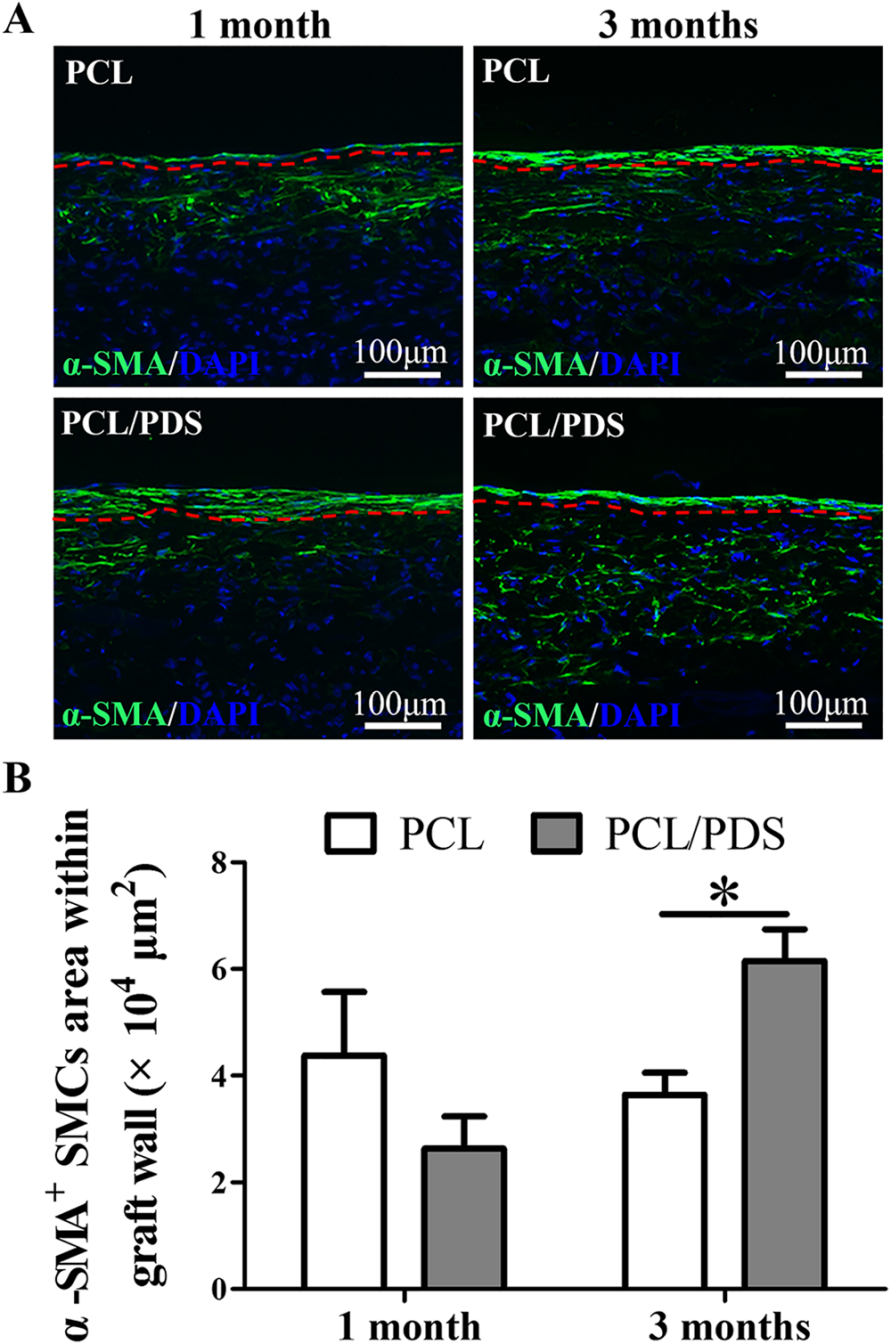
Synthetic smooth muscle regeneration of grafts at 1 and 3 months after implantation. (**A**) Longitudinal sections were stained with anti-smooth muscle actin (α-SMA) antibody to identify the regeneration of synthetic phenotype SMCs. (**B**) The synthetic phenotype SMCs area within the graft walls were also calculated based on α-SMA staining. *p < 0.05. The region below the red line represented graft wall.

**Figure 5:**
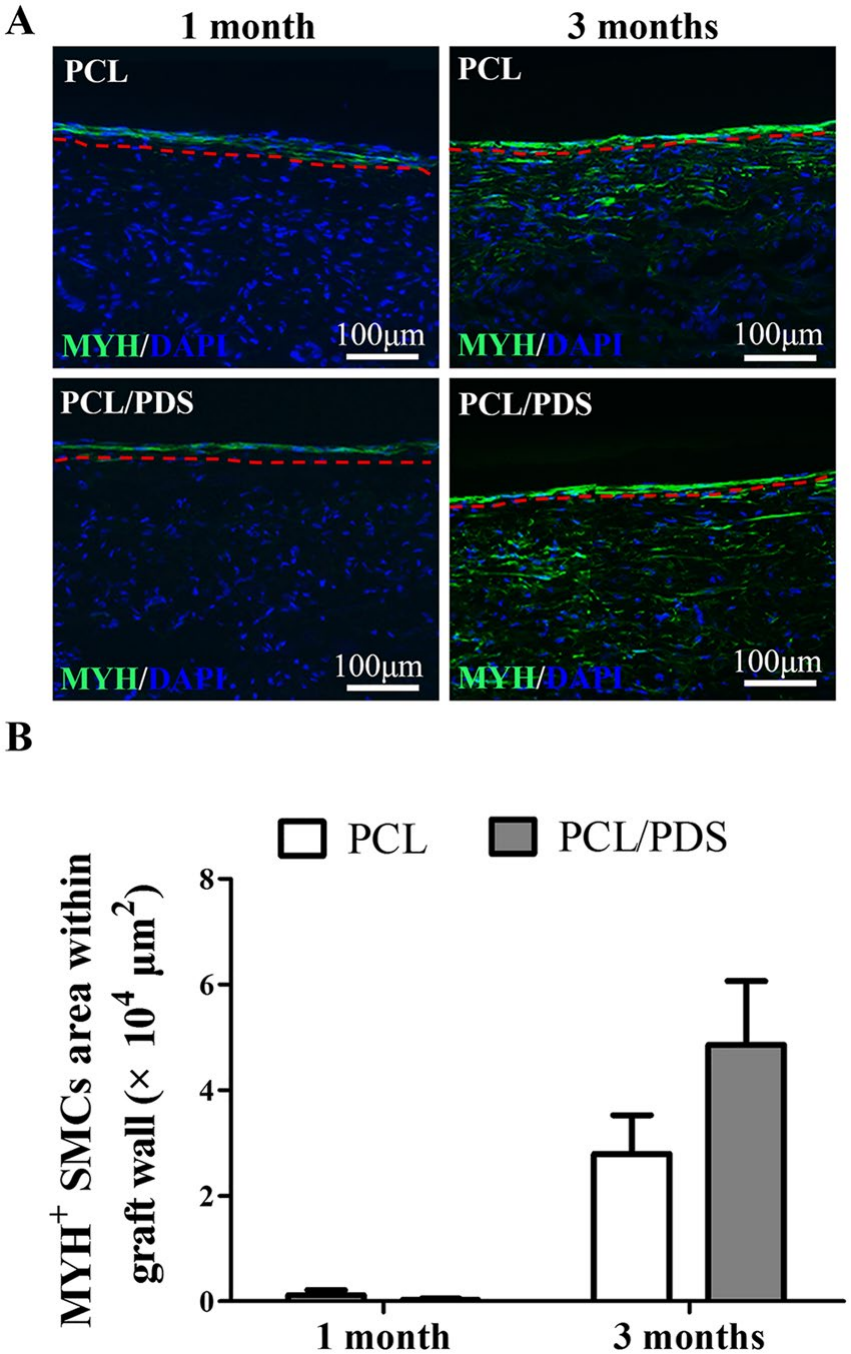
Contractile smooth muscle regeneration of explanted grafts at 1 and 3 months after implantation. (**A**) Longitudinal sections were stained with anti-smooth muscle myosin heavy chain I (MYH) antibody to identify the regeneration of contractile phenotype SMCs. (**B**) The contractile phenotype SMCs area within grafts wall were also calculated based on MYH staining. The region below the red line represented graft wall.

The regeneration of synthetic and contractile phenotype SMCs was identified by staining the longitudinal sections with α-SMA (Supplementary Fig. S11A and Fig. 4A) and MYH antibody (Supplementary Fig. S12A and Fig.5A), respectively. At 1 month, the coverage of α-SMA^+^ layer in PCL/PDS grafts (93.34 ± 3.08%) was slightly higher than that of PCL (81.77 ± 6.76%) (Supplementary Fig. S11B), but the coverage of MYH^+^ layer in PCL/PDS grafts (77.44 ± 5.23%) was significantly (*p* < 0.05) higher than that of PCL (51.42 ± 7.66%) (Supplementary Fig. S12B). Previous study confirmed that ECs could actively promote SMCs differentiation from synthetic to contractile phenotype^25^. We believed that the rapid endothelialization of PCL/PDS grafts may lead to the increased coverage of contractile SMCs on the PCL/PDS grafts. At 3 months, although the coverage of α-SMA^+^ and MYH^+^ layers was enhanced in both grafts, two types SMCs layer coverage on PCL/PDS grafts was still better than that those on PCL grafts (Supplementary Fig. S11B; Supplementary Fig. S12B).

As can be seen from Fig. 4A and Fig. 5A, the SMCs regeneration within grafts was different between the PCL and PCL/PDS grafts. At 1 month, there were more α-SMA^+^ SMCs in PCL grafts wall than those in PCL/PDS grafts, while MYH^+^ SMCs could hardly be identified in both grafts. After 3 months, the α-SMA^+^ cells within the PCL/PDS grafts were uniformly distributed in the area close to the lumen (Fig.4A; Supplementary Fig. S11A), with an occupied area about 1.5 times larger than that in the PCL grafts (Fig. 4B *, p*  < 0.05). Similarly, the area of MYH^+^ SMCs within PCL/PDS grafts was about 2 times larger than that in the PCL grafts (Fig. 5B, *p* = 0.196). At 1 month, the explanted PCL and PCL/PDS grafts are comparable in terms of fiber density. However, at 3 months, the fiber density of PCL/PDS grafts was lower than that of PCL grafts (Supplementary Fig. S13), which indicated the degradation of PDS fibers. The degradation of PDS fibers within hybrids grafts provide more space to promote smooth muscle regeneration within PCL/PDS grafts.

Wang’s group investigated the effect of pore size in PGS porous scaffold on SMC organization. They found that pores of 25–32 μm increased SMCs alignment, production of elastin and collagen^26^, which indicated SMCs tended to survive in the scaffolds with pores of 25–32 μm. After degradation *in vivo* for 12 weeks, the pore size of PCL/PDS scaffolds was 31.04 ± 0.87 μm, which was just within the above size range. This may be another reason for improved smooth muscle regeneration within PCL/PDS grafts. In fact, pore size, fiber diameter^6^ and alignment^27^ played an important role in determining the vascular regeneration. Therefore, after completely degradation of one component in hybrid grafts, the structure of residual grafts is an important factor that should be taken into consideration for designing the hybrid grafts.

### ECM remodeling

**Figure 6:**
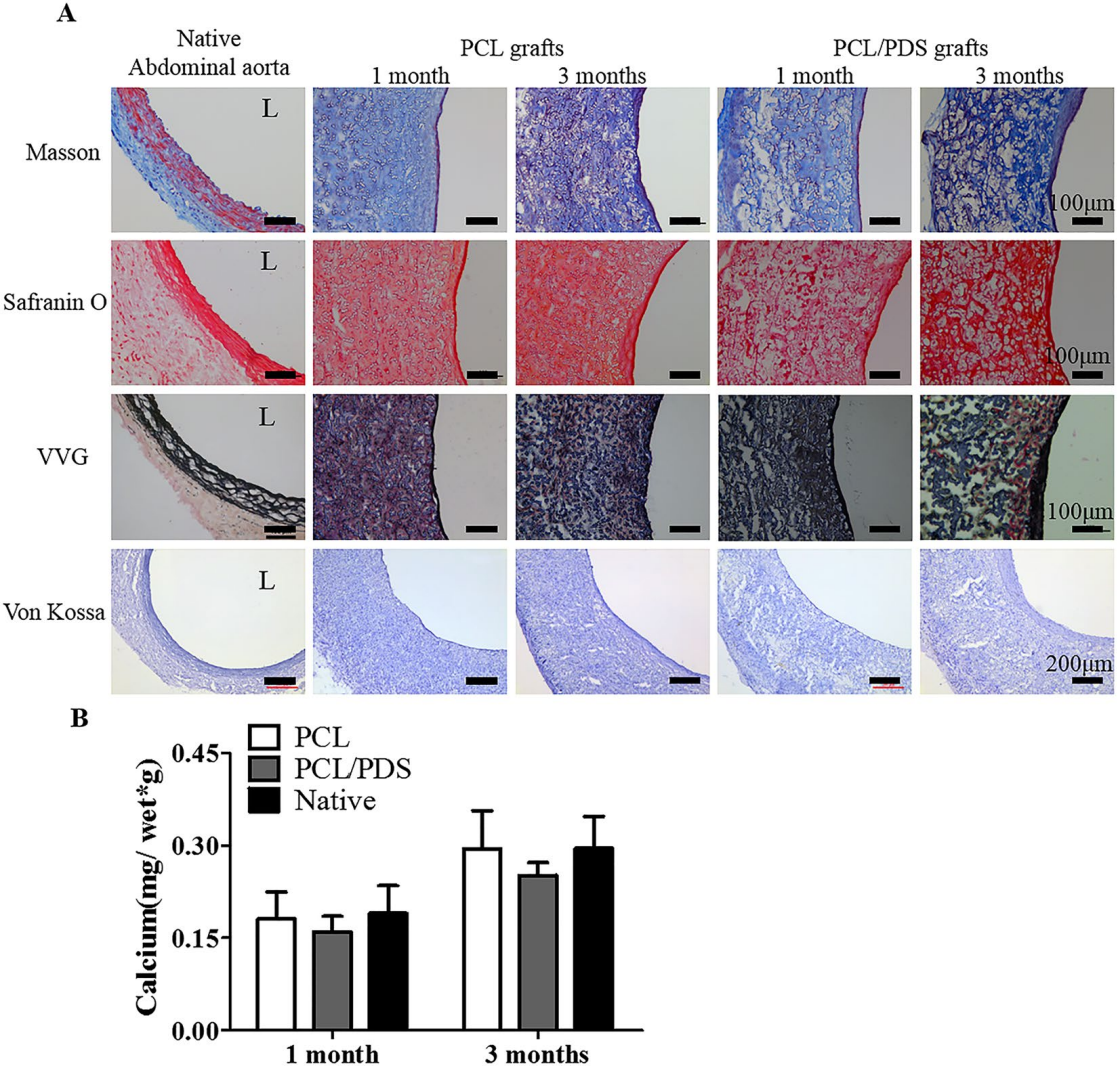
ECM reconstruction of explanted grafts at 1 and 3 months after implantation. (**A**) The cross sections were stained with Masson’s Trichrome, Safranin O, Verhoeff-Van Gieson (VVG) and Von Kossa to identify the presence of collagen, glycosaminoglycan, elastic fibers and calcification, respectively. (**B**) Atomic absorption spectrophotometer showed that the calcium concentrations of the explanted grafts were close to native abdominal aorta.

ECM remodeling is vital for vascular regeneration. In this study, the Masson, Safranin O, Verhoeff-van Gieson (VVG) and Von Kossa staining were performed to detect the collagen, elastin fibers, glycosaminoglycan and calcification formation, respectively. The neointima of the two types of grafts both showed ECM deposition, which was similar to the SMCs layer of native abdominal aorta (Fig. 6A). However, the ECM remodeling was different in the PCL and PCL/PDS graft walls. Compared to the PCL grafts, the collagen, glycosaminoglycan and elastin fibers within PCL/PDS graft walls showed spare distribution (Fig. 6A). The large pore generated by PDS degradation has not been completely filled with ECM at 3 months, which corresponds to the rapid degradation of PDS fibers that was faster than the ECM remodeling. Hence, an ideal scaffold used for tissue engineering should hold an appropriate degradation rate which well matches with that of ECM formation^28^. Calcification remains one of the major obstacles restricting the translation of vascular grafts for arterial repair^29^. Von Kossa staining and the calcium quantification showed that there was no calcification in the wall of PCL/PDS grafts (Fig. 6A,B).

In this study, a hybrid vascular graft composed of PCL and PDS was prepared via electrospinning with a side-by-side dual spinneret method. The incorporation of PDS improved mechanical properties, hydrophicity of the hybrid grafts compared to PCL grafts. The PDS fibers completely degraded within 12 weeks *in vitro/vivo*, which resulted in the increased pore size of PCL/PDS grafts. The hybrid grafts exhibited good blood compatibility and patency in rat abdominal aorta model, similar to PCL grafts. The PCL/PDS grafts could resist aneurysm formation despite degradation of PDS fibers. Endothelialization and contractile phenotype SMCs coverage was significantly better in the PCL/PDS grafts. The degradation of PDS fibers provided extra space for improving SCMs regeneration within hybrid graft walls. In summary, our hybrid PCL/PDS grafts may be a promising off-the-shelf vascular graft candidate and are worthy of further evaluation with long term.

## Methods

### Materials

PCL pellets (*M*n = 80,000) were purchased from Sigma (St Louis, USA). PDS pellets were purchased from Tianjin Dongnanhesheng Medical Technologies Co., Ltd (Tianjin, China). 1, 1, 1, 3, 3, 3-fluoro-2-propanol (HFIP) was purchased from Aladdin (Shanghai, China). Methanol, chloroform and alcohol were obtained from Tianjin Chemical Reagent Company (Tianjin, China). 3, 3′-dioctade-cyloxacarbocyanine perchlorate (DiO) and 1, 1′-dioctadecyl-3, 3, 3′, 3′-tetramethylindocarbocyanine perchlorate (DiI) were products of Molecular Probes (Eugene, OR). Triton X-100 was purchased from Alfa Aesar (London, England). Sprague Dawley (SD) rats (male, weight 280–320 g) were purchased from the Laboratory Animal Center of the Academy of Military Medical Sciences (Beijing, China). All animal studies were performed under the guidelines set by the Tianjin Committee of Use and Care of Laboratory Animals, and the overall project protocols were approved by the Animal Ethics Committee of Nankai University.

### Fabrication of hybrid PCL/PDS fibrous vascular grafts

The hybrid PCL/PDS vascular grafts were prepared by co-electrospinning. A 25% w/v solution of PCL was prepared in a 5:1 (V/V) mixture of chloroform and methanol by stirring overnight. PDS was dissolved in the HFIP with stirring at room temperature for 6 h to obtain 20% w/v solution. Two 10-mL syringes were filled with PCL or PDS solution and connected to a 21 G blunt-ended needle that served as the charged spinneret. The apparatus consists of a syringe pump (Cole Parmer, Vernon Hills, IL), a high-voltage generator (DWP503-1AC, Dong-Wen High Voltage power supply Factory, Tianjin, China) and a rotating steel mandrel (2.0 mm in diameter) as collector. The flow rate of PCL was set at 8 mL/h. The flow rate of PDS was set at 4 mL/h, 6 mL/h, 10 mL/h and 16.67 mL/h, respectively, to fabricate hybrid PCL/PDS grafts with different ratios. The voltages between the needle tip and the rotating mandrel were set as 11 kV for PCL and 15 kV for PDS. The distance between the needle tip and collector were 25 cm for PCL and 15 cm for PDS. The pure PCL grafts and pure PDS grafts were regarded as control and prepared by the same method as described above. The obtained electrospun grafts were vacuum-dried over 48 h at room temperature before further treatment.

### Fiber morphology and distribution

The cross-section and lumen surface of grafts were mounted on an aluminum foil and sputter coated with gold and palladium. Scanning electron microscope (SEM, HITACHI, X-650, Japan) at an accelerating voltage of 15 kV was used to observe the morphology of grafts structure. Based on the SEM images, fiber diameter was analyzed using Image-Pro Plus software (IPP). The pore diameter was calculated according to the method described by Dong *et al*.^30^. At least six pores per image, five images per sample and three samples per group were included to obtain the calculation.

To visualize the distribution of the PCL and PDS fibers, two different fluorescent dyes were incorporated into each fibers of the graft. DiI (1 mg/mL, orange–red fluorescent) was added to PCL solution and DiO (1 mg/mL, green fluorescent) was added to PDS solution. After co-electrospinning, the PCL/PDS grafts were visualized using a laser scanning confocal microscope (CLSM; Zeiss LSM710).

### The ratio of PCL and PDS in the hybrid grafts

The weighted hybrid PCL/PDS grafts (1.1 cm in length) were soaked in 10 ml of chloroform in a 15 ml centrifugal tube, and kept in a shaker at room temperature for 12 h. The chloroform was changed every 4 h to completely leach the PCL. After leaching, the grafts were vacuum-dried over 48 h at room temperature. Then the weight of grafts was measured. The ratio of PCL and PDS in the hybrid graft was determined as follows: the ratio of PCL and PDS = (weight of graft before leaching−weight of graft after leaching)/weight of graft after leaching.

### Mechanical test

Mechanical properties of the grafts in transverse directions were measured on a tensile-testing machine with a load capacity of 100 N (Instron-3345, Norwood, MA). Grafts with 0.3 cm in length were fixed on two steel rings which were clamped by machine chuck, and then pulled radially at a rate of 10 mm/min until rupture. Tensile strength and ultimate elongation at break were measured. Young’s modulus was obtained by measuring the slope of the stress-strain curve in the elastic region. These mechanical tests were performed in triplicate according to the ANSI guidelines.

### *In vivo* implantation in rats

Twenty SD rats were anesthetized with intraperitoneal injection of chloral hydrate (330 mg/kg body weight). Heparin (100 unit/kg) was used for anticoagulation by tail vein injection before surgery. A midline laparotomy incision was made and the abdominal aorta was isolated, clamped, and transected. The PCL, PCL/PDS and PDS grafts (1.1 cm in length) were sewed in an end-to-end fashion with 8 interrupted stitches using 9-0 monofilament nylon sutures (Yuan Hong, Shanghai, China). The wound was closed with 3-0 monofilament nylon sutures. No anticoagulation drug was administered after surgery. The grouping of animals was based on the type of scaffolds and duration of observation for 1 and 3 months.

### Color Doppler Ultrasound

At the predetermined time points (1 and 3 months), the patency and blood velocity of the grafts was visualized by high-resolution ultrasound (Vevo 2100 System, Canada) after the rats were anesthetized with isoflurane.

### **H**istological analysis of the explanted vascular grafts

At each time point, the rats were sacrificed by injection of over-dose urethane. The grafts were explanted and perfused with saline before they were harvested. The explants were cut into two parts from the middle. One part was snap-frozen in optimal cutting temperature (OCT) Compound (Tissue Tek) for frozen cross-section. The other part was longitudinally cut into three pieces. One piece was observed by stereomicroscope, and then snap-frozen in OCT for longitudinal section. Other two pieces were prepared for SEM and *En face* immunofluorescence staining.

The samples embedded in OCT were cut into 6 μm in thickness. Subsequently, the sections were stained with hematoxylin and eosin (H&E), Masson’s trichrome, Safranin O, Verhoeffe-Van Gieson (VVG) and Von Kossa. Images were observed under upright microscope (Leica DM3000).

For immunofluorescent staining, the frozen sections were fixed in acetone at −20 °C for 10 min, air-dried, and rinsed once with 0.01 mM PBS. Then slides were incubated in 5% normal goat serum (Zhongshan Golden Bridge Biotechnology, China) for 45 min at 4 °C. To stain intracellular antigens, 0.1% Triton-PBS was used to permeate the membrane before incubation with serum. The sections were incubated with the following primary antibodies overnight at 4 °C: Mouse anti- CD31 (1:100, Abcam, USA), mouse anti-alpha smooth muscle actin (a-SMA, 1:100, Boster, China), mouse anti-smooth muscle myosin heavy chain I (MYH, 1:500, Abcam, USA). After rinsing with PBS for five times, Alexa Fluor 488 goat anti-mouse IgG (1:200, Invitrogen) was applied for 2 h at room temperature. The nuclei were counterstained with 4′,6-diamidino-2-phenylindole (DAPI) containing mounting solution (Dapi Fluoromount G, Southern Biotech, England). The sections without incubation with primary antibody were used as negative controls. Slides were observed under a fluorescence microscope (Zeiss Axio Imager Z1, Germany), and the images were acquired with a digital camera (Axio Cam MRm, Germany).

To quantify the cell density of graft walls, the cross-sections of grafts were stained with DAPI. Ten high-magnification DAPI images per sample, five samples per group at 1month and four samples per group at 3 months were included to quantify the cell density.

To prepare samples for SEM, the explanted grafts were fixed with 2.5% glutaraldehyde for 12 h, and dehydrated in ascending series of ethanol. They were affixed onto aluminum stubs with carbon tape, sputter-coated with gold, and observed by SEM.

### *En face* immunofluorescence microscopy

The explanted grafts were fixed with 2% paraformaldehyde for 30 min at room temperature. Then the samples were washed with PBS, blocked with 2% bovine serum albumin for 2 h at 4 °C, and incubated with primary antibody against endothelial cells marker CD31 overnight at 4 °C, and then incubated with Alexa-Fluor 488 labeled secondary antibodies. To prepare stained whole mounts for imaging, segments were mounted on glass slides, flattened under a coverslip. The luminal surface of the explanted grafts was imaged under a confocal laser scanning microscope.

### Statistical Analysis

GraphPad Prism Software Version 5.0 (San Diego, CA, USA) was used for statistical analysis. Single comparisons were made with a paired Student’s t-test. Multiple comparisons were carried out using one-way ANOVA and Tukey’s post-hoc analysis. The minimum significance level was set at *p < 0.05 and ^#^ *p* < 0.001. All data were shown as mean ± standard error of the mean (SEM). Other Parts are available in the Supporting Information.

## Electronic supplementary material

Supporting Information

## References

[1] Roth GA (2015). Demographic and epidemiologic drivers of global cardiovascular mortality. New England Journal of Medicine.

[2] Zilla P, Bezuidenhout D, Human P (2007). Prosthetic vascular grafts: wrong models, wrong questions and no healing. Biomaterials.

[3] Tang Z, Callaghan J, Hunt J (2005). The physical properties and response of osteoblasts to solution cast films of PLGA doped polycaprolactone. Biomaterials.

[4] de Valence S (2012). Long term performance of polycaprolactone vascular grafts in a rat abdominal aorta replacement model. Biomaterials.

[5] Pektok E (2008). Degradation and healing characteristics of small-diameter poly (ε-caprolactone) vascular grafts in the rat systemic arterial circulation. Circulation.

[6] Wang Z (2014). The effect of thick fibers and large pores of electrospun poly (ε-caprolactone) vascular grafts on macrophage polarization and arterial regeneration. Biomaterials.

[7] Zheng W (2012). Endothelialization and patency of RGD-functionalized vascular grafts in a rabbit carotid artery model. Biomaterials.

[8] Wu W, Allen RA, Wang Y (2012). Fast-degrading elastomer enables rapid remodeling of a cell-free synthetic graft into a neoartery. Nature medicine.

[9] Baker BM (2008). The potential to improve cell infiltration in composite fiber-aligned electrospun scaffolds by the selective removal of sacrificial fibers. Biomaterials.

[10] Baker BM, Nerurkar NL, Burdick JA, Elliott DM, Mauck RL (2009). Fabrication and modeling of dynamic multipolymer nanofibrous scaffolds. Journal of Biomechanical Engineering.

[11] Ray J, Doddi N, Regula D, Williams J, Melveger A (1981). Polydioxanone (PDS), a novel monofilament synthetic absorbable suture. Surgery, gynecology & obstetrics.

[12] Im JN (2007). *In vitro* and *in vivo* degradation behaviors of synthetic absorbable bicomponent monofilament suture prepared with poly (p-dioxanone) and its copolymer. Polymer degradation and stability.

[13] Zamiri P (2010). The biocompatibility of rapidly degrading polymeric stents in porcine carotid arteries. Biomaterials.

[14] Schwarcz T, Nussbaum M, Ellinger J, Kim D, Greisler H (1986). Prostaglandin content of tissue lining vascular prostheses. Current surgery.

[15] McClure M, Sell S, Ayres C, Simpson D, Bowlin G (2009). Electrospinning-aligned and random polydioxanone–polycaprolactone–silk fibroin-blended scaffolds: geometry for a vascular matrix. Biomedical Materials.

[16] Tan Z, Wang H, Gao X, Liu T, Tan Y (2016). Composite vascular grafts with high cell infiltration by co-electrospinning. Materials Science and Engineering: C.

[17] Hasan A (2014). Electrospun scaffolds for tissue engineering of vascular grafts. Acta biomaterialia.

[18] Pham QP, Sharma U, Mikos AG (2006). Electrospun poly(epsilon-caprolactone) microfiber and multilayer nanofiber/microfiber scaffolds: characterization of scaffolds and measurement of cellular infiltration. Biomacromolecules.

[19] Kai, W. *et al*. Enhanced Vascularization in Hybrid PCL/Gelatin Fibrous Scaffolds with Sustained Release of VEGF. *Biomed Research International* **2015** (2014). 10.1155/2015/865076 PMC4390103 25883978

[20] Li Q (2013). Functionalization of the surface of electrospun poly(epsilon-caprolactone) mats using zwitterionic poly(carboxybetaine methacrylate) and cell-specific peptide for endothelial progenitor cells capture. Materials Science & Engineering C.

[21] Kai, W. *et al*. Three-Layered PCL Grafts Promoted Vascular Regeneration in a Rabbit Carotid Artery Model. *Macromolecular Bioscience* (2016). 10.1002/mabi.201500355 26756321

[22] Chung S, Ingle NP, Montero GA, Kim SH, King MW (2010). Bioresorbable elastomeric vascular tissue engineering scaffolds via melt spinning and electrospinning. Acta Biomaterialia.

[23] Heo Y, Shin YM, Yu BL, Lim YM, Shin H (2015). Effect of immobilized collagen type IV on biological properties of endothelial cells for the enhanced endothelialization of synthetic vascular graft materials. Colloids & Surfaces B Biointerfaces.

[24] Ren X (2015). Surface modification and endothelialization of biomaterials as potential scaffolds for vascular tissue engineering applications. Chemical Society Reviews.

[25] Brown DJ (2005). Endothelial cell activation of the smooth muscle cell phosphoinositide 3-kinase/Akt pathway promotes differentiation. Journal of Vascular Surgery.

[26] Kee-Won Lee DBS (2011). Yadong Wang. Substantial expression of mature elastin in arterial constructs. Proceedings of the National Academy of Sciences of the United States of America.

[27] Zhu M (2015). Circumferentially aligned fibers guided functional neoartery regeneration *invivo*. Biomaterials.

[28] Seyednejad H (2011). An Electrospun Degradable Scaffold Based on a Novel Hydrophilic Polyester for Tissue-Engineering Applications. Macromolecular Bioscience.

[29] Tara S (2014). Well-organized neointima of large-pore poly(L-lactic acid) vascular graft coated with poly(L-lactic-co-ε-caprolactone) prevents calcific deposition compared to small-pore electrospun poly(L-lactic acid) graft in a mouse aortic implantation model. Atherosclerosis.

[30] Wang S, Zhang Y, Wang H, Yin G, Dong Z (2009). Fabrication and properties of the electrospun polylactide/silk fibroin-gelatin composite tubular scaffold. Biomacromolecules.

