# Supplementary material for: Small-diameter hybrid vascular grafts composed of polycaprolactone and polydioxanone fibers
Source: Sci Rep. 2017 Jun 15;7:3615. doi: 10.1038/s41598-017-03851-1 (PMC5472623; doi:10.1038/s41598-017-03851-1)
Supplement: Supplementary file 1 — Supporting Information [file 41598_2017_3851_MOESM1_ESM.pdf]

Supporting Information

Small-diameter hybrid vascular grafts composed of polycaprolactone and polydioxanone fibers

Yiwa Pan[1,*], Xin Zhou[1,*], Yongzhen Wei[1], Qiuying Zhang[1], Ting Wang[2], Meifeng Zhu[1], Wen Li[1], Rui Huang[1], Ruming Liu[1], Jingrui Chen[3], Guanwei Fan[3], Kai Wang[1], Deling Kong[1], Qiang Zhao[1]

[1]Key Laboratory of Bioactive Materials, Ministry of Education, Nankai University, Tianjin 300071, China

[2]Urban Transport Emission Control Research Centre, College of Environmental Science and Engineering, Nankai University, Tianjin 300071, China

[3]Center for Research and Development of Chinese Medicine, Tianjin State Key Laboratory of Modern Chinese Medicine, Tianjin University of Traditional Chinese Medicine, Tianjin 300193, China

[*]Authors contributed equally to this work

Corresponding authors:
Dr. Kai Wang: 013053@nankai.edu.cn
Dr. Qiang Zhao: qiangzhao@nankai.edu.cn

1. Experimental Section

1.1 Electrospinning of PCL/PDS scaffolds

The hybrid PCL/PDS scaffolds were prepared by co-electrospinning. A 25% w/v solution of PCL was prepared in a 5:1 (V/V) mixture of chloroform and methanol by stirring overnight. PDS was dissolved in the 1,1,1,3,3,3-fluoro-2-propanol (HFIP) with stirring at room temperature for 6 h to obtain 20% w/v solution. Two 10-mL syringes were filled with PCL or PDS solution and connected to a 21 G blunt-ended needle that served as the charged spinneret. The apparatus consisted of a syringe pump (Cole Parmer, Vernon Hills, IL), a high-voltage generator (DWP503-1AC, Dong-Wen High Voltage power supply Factory, Tianjin, China) and a rotating mandrel (15cm in diameter) as collector. The flow rate of PCL and PDS was set at 8 mL/h and 6mL/h, respectively. The voltages between the needle tip and the rotating mandrel were set as 11 kV for PCL and 15 kV for PDS. The distance between the needle tip and collector were 25 cm for PCL and 15 cm for PDS. The obtained electrospun scaffolds were vacuum-dried over 48 h at room temperature before further treatment. The PCL films and PDS films were regarded as control for following experiments.

1.2 Water contact angle (WCA) measurements

The sessile drop method was used to measure WCA at room temperature through an optical contact goniometer (Harke-SPCA, Beijing, China). The scaffolds were pasted on glass slides and fixed onto the sample holder. Each measurement was performed using a 10 µL drop of ddH_2O on the surfaces of the scaffolds after 20s. The average values of WCA were averaged based on three values at different positions of the sample surface.

1.3 *In vitro* hemocompatibility evaluation

For clotting time assay, the PCL, PCL/PDS and PDS scaffolds were cut into circular disks (1 cm in diameter). Then 1 mL of human plasma was put into a tapered tube, and a circular sample was added. A tapered tube without addition of the sample was used as control. The plasma was allowed to incubate for 1 h at 37 ℃. The full human plasma activated partial thromboplastin time (APTT), thromboplastin time (TT) and prothrombin time (PT) of each sample were measured on an automatic SYSMEX CA-7000 coagulation analyzer. The data were averaged from measurements on three specimens.

For hemolysis assay, 10 ml of 0.2% (v/v) citrated rat blood in 0.9 % NaCl was added to a 15 ml centrifugal tube with circular samples (1 cm in diameter, n = 3) on the bottom, and kept in a shaking bath at 37 ℃ for 2 h. After centrifugation (1,000 rpm, 5 min), the supernatant from each sample was transferred into a 96-well plate and measured by a Bio-Rad Microplate Reader (iMark, Bio-Rad, USA) at 540 nm. As a positive control for hemolysis, 200 μL of citrated blood was diluted in 10 mL of distilled water; meanwhile 200 μl of citrated rabbit blood was diluted in 10 mL of 0.9 % NaCl served as a negative control. The hemolysis ratio was calculated according to the following equation:

$$\text{Hemolysis \%} = (OD_{\text{Sample}} - OD_{\text{Negative Control}})/OD_{\text{Positive Control}} \times 100$$

For fibrinogen (FGN) adsorption assay, scaffolds with an appropriate size were placed into a 96-well plate (n = 3). 100 μL of 30μg/mL human FGN in PBS was added into each well and incubated for 1 h at 37 ℃. After the sample was washed with PBS (5 min × 3 times), the enzyme-linked immunosorbent assay (ELISA) was carried out with a Human Fibrinogen ELISA Kit according to the literature. The OD value of substrate solution was read at 450 nm with a microplate reader.

1.4 *In vitro* biodegradation evaluation

Each of the 1 cm×2.5 cm sterilized and known weight electrospinning scaffolds (n = 5) was

immersed in 10 mL of phosphate-buffered saline (PBS) solution at pH 7.4 in sterilized capped containers. Sodium azide (0.1% (w/w)), penicillin (100 U/mL), and streptomycin (100 μg/mL) were added to the solution as the antimicrobials to prevent bacterial growth. These containers were then incubated at 37 ℃with agitation. At the predetermined time points, the scaffolds were taken out from the sterile solution, rinsed with ddH_2O, dried to constant weight under vacuum and weighed. The physical appearance of the scaffolds before and after degradation in PBS was observed by a stereomicroscope. The extent of *in vitro* biodegradation was expressed as the percentage of the weight of dried scaffolds after PBS treatment to the original weight. In addition, the changes of fibers morphology of scaffolds degraded *in vitro* were evaluated by scanning electron microscope (SEM, HITACHI, X-650, Japan). Based on the SEM images, fiber diameter was analyzed using Image-Pro Plus software. The pore diameter was calculated according to the method described by Dong *et al* [1]. At least six pores per image, three images per sample and three samples per group were included to obtain the calculation.

1.5 *In vivo* biodegradation evaluation

Electrospinning scaffolds were cut into round discs (1cm in diameter). The Sprague Dawley (SD) rats (male, weight 250–300g) were anesthetized with intraperitoneal injection of chloral hydrate (330 mg/kg body weight). The samples (n = 4) were implanted subcutaneously at one side of the backbone. The grouping of animals was based on the type of scaffolds and duration of observation for 2, 4 and 12 weeks. Upon explantation, the physical appearance of explanted samples was firstly observed by a stereomicroscope. Then the explanted samples were treated with 1.25 wt% trypsin to remove the surrounding tissues for SEM.

1.6 The degradation analysis of explanted grafts

The explanted grafts were embedded in OCT, and cut into 6 μm in thickness. Subsequently, the sections rinsed twice with 0.01 mM PBS and sealed with coverslips using PBS/glycerin (v/v=1:1). Slides were observed under stereomicroscope (Leica M165 C) using Hoffmann modulation phase contrast (HMC) method.

1.7 Mechanical test of the explanted PCL/PDS vascular grafts

At each time point, PCL/PDS vascular grafts were explanted and the surrounding tissues were removed under a stereoscopic microscope. Then the explanted PCL/PDS grafts were cut into vascular graft rings with 0.3 cm in length. Transverse mechanical test of the grafts was measured on a tensile-testing machine with a load capacity of 100 N (Instron-3345, Norwood, MA). The graft rings were fixed on a special two-pin frame which were clamped by machine chuck and then pulled radially at a rate of 10 mm/min until rupture. Tensile strength was measured. Young's modulus was obtained by measuring the slope of the stress-strain curve in the elastic region. The native abdominal aorta as control group was tested with the same protocol. These mechanical tests were performed in triplicate.

2. Supplementary Figure

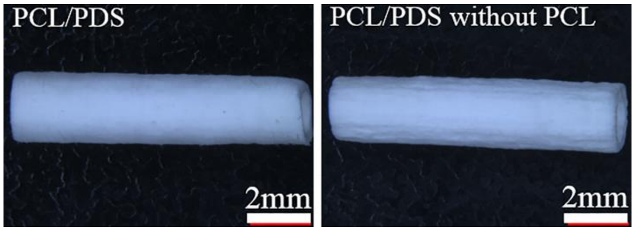

Fig. S1 The gross morphology of the PCL/PDS grafts before and after washed by chloroform to leach PCL fibers.

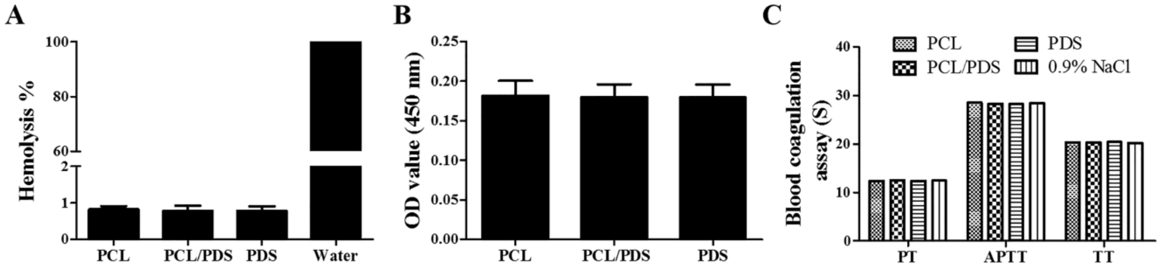

Fig. S2 Hemocompatibility evaluation of vascular grafts. A: Hemolysis rate of all scaffolds was about 1%, which was much lower thanthe accepted threshold value of 5% (GB/T 16886 and ISO 10993). B: The adsorbed fibrinogen on scaffolds was tested by a HumanFibrinogen ELISA Kit. The result showed fibrinogen absorption on PCL/PDS and PDS scaffolds was equal to that on PCL scaffolds. C: Blood coagulation test of PCL, PCL/PDS and PDS scaffolds. Prothrombin time (PT), activated partial thromboplastin time (APTT) and thrombin time (TT) was regard as parameter to detect the clotting time of the three types of scaffolds. The clotting time of human plasma without any interference was also tested as control group. The result showed the clotting time of the three types of scaffolds was the same as the control group, which indicated that three types of scaffolds did not cause actu coagulation.

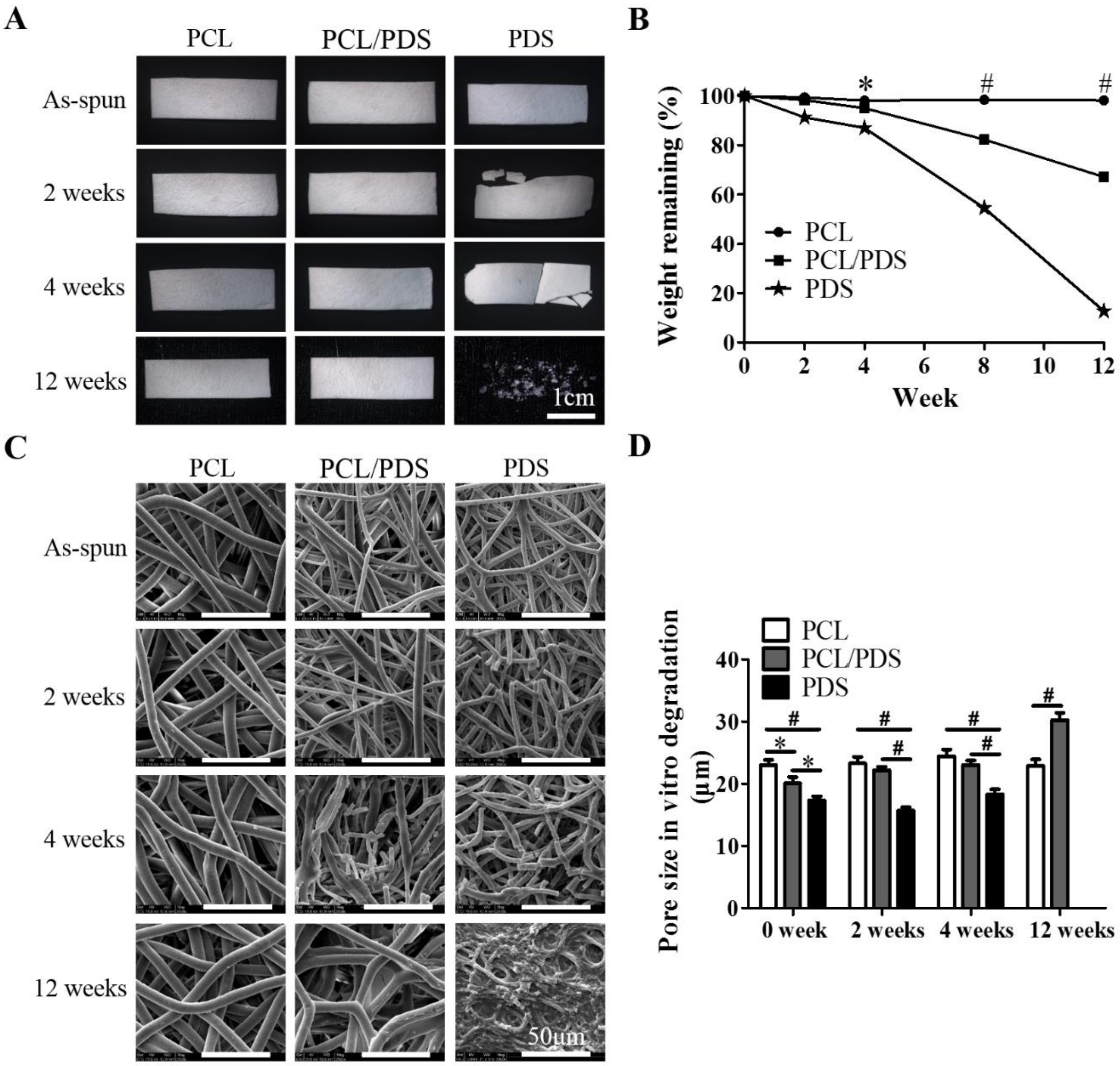

Fig. S3 The degradation of the electrospun PCL, PCL/PDS and PDS scaffolds (1 cm×2.5 cm) after 2, 4 and 12 weeks of immersion in PBS. A: The change of physical appearance of scaffolds was observed by stereomicroscope. B: Quantitative analysis on the weight remaining of scaffolds. C: The morphology of scaffolds after degradation in PBS was observed by SEM. D: The average pore size of scaffolds were calculated based on SEM images. $^{*}p < 0.05$, $^{\#}p < 0.001$.

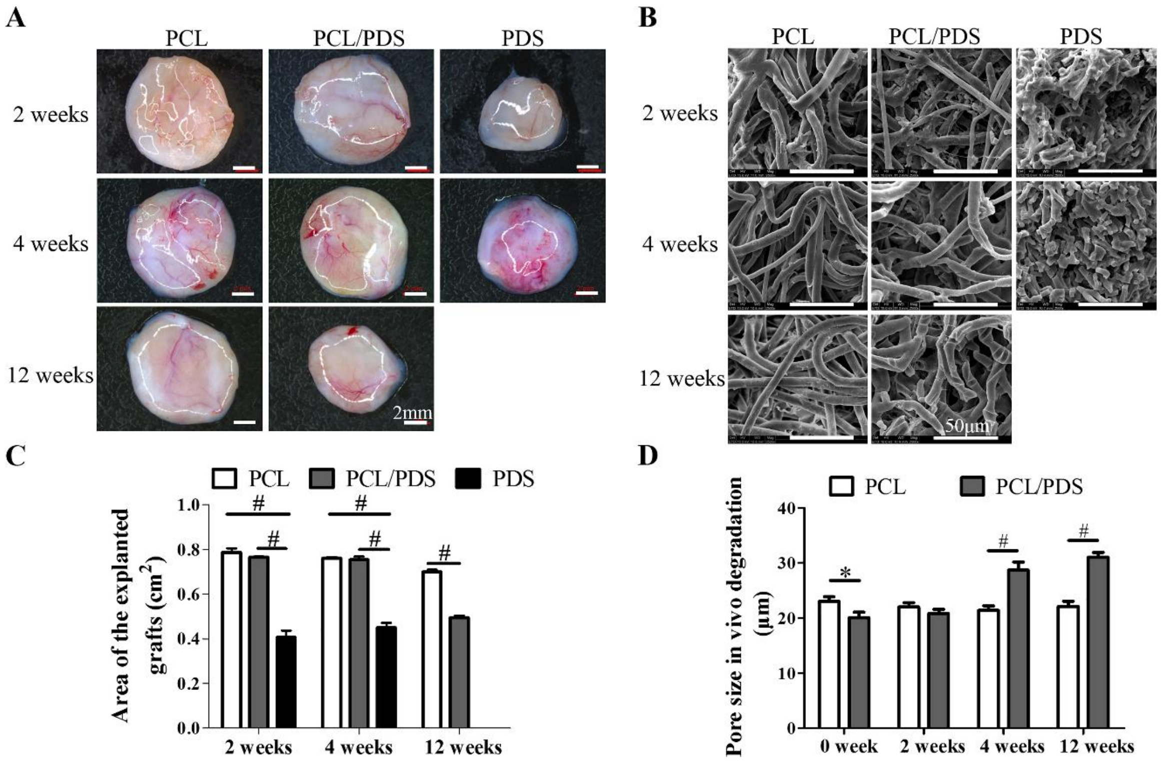

Fig. S4 The degradation of the electrospun PCL, PCL/PDS and PDS scaffolds (1cm in diameter) after 2, 4 and 12 weeks of subcutaneous implantation in rat. A: The change of gross morphology appearance of explanted scaffolds was observed by stereomicroscope. B: Quantitative analysis on the area of explanted scaffolds. C: The morphology of scaffolds after degradation *in vivo* was observed by SEM. D: The average pore size of scaffolds were calculated based on SEM images. $^{*}p < 0.05$, $^{\#}p < 0.001$.

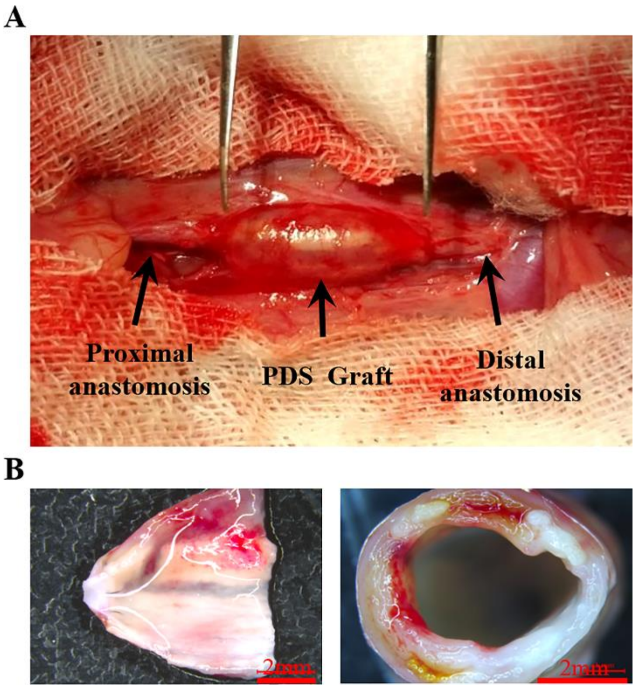

Fig. S5 Aneurysmal dilatation of PDS grafts at 1 month after implantation. A: The PDS grafts showed aneurysm formation in rat abdominal aorta. B: The stereomicroscope images showed the explanted PDS grafts displayed partly rupture in the grafts wall and obvious dilation.

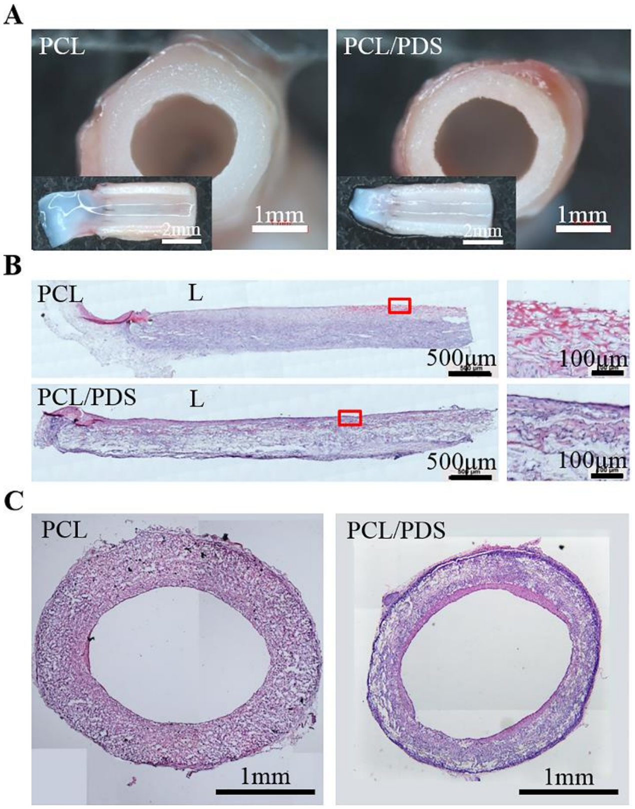

Fig. S6 Evaluation of the patency and intimal hyperplasia of explanted grafts at 1 month after implantation. A: The lumen of the explanted grafts was smooth and free of thrombus under stereomicroscope. B: Longitudinal sections were stained with H&E to identify the neointima formation. C: Representative H&E staining of cross sections of explanted grafts. L: lumen; Red arrows: suture site.

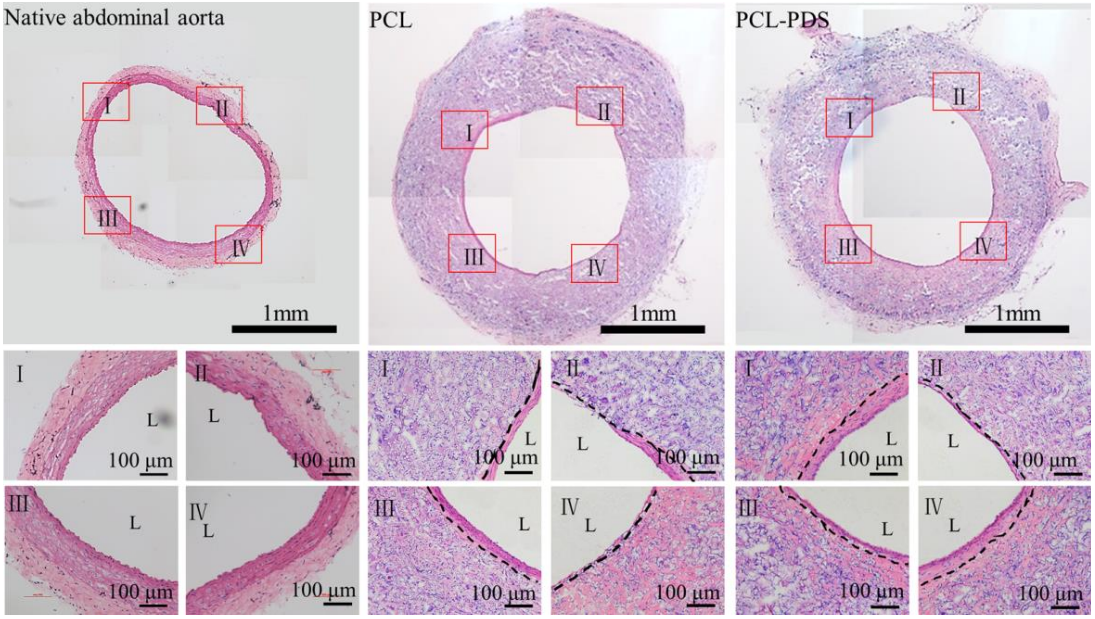

Fig. S7 The magnified H&E images of different regions of cross sections of explanted grafts after implantation for 3 months. The black dotted lines denoted the neointimal boundary of the grafts.

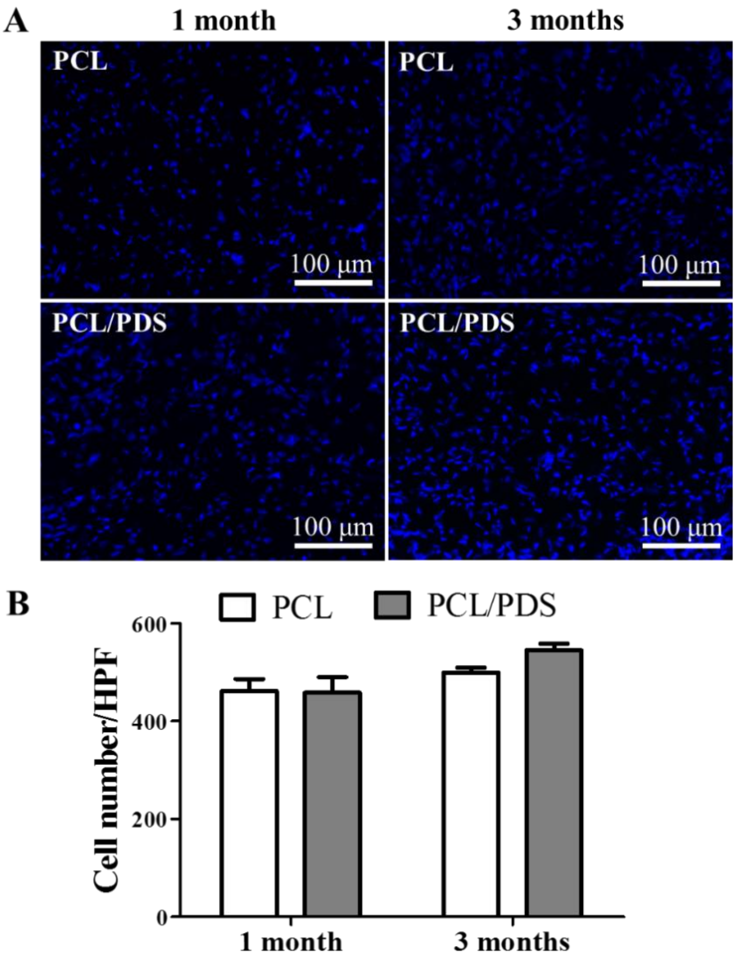

Fig. S8 The cell density of PCL and PCL/PDS grafts wall after 1 and 3 months in rat abdominal aorta implantation. In order to quantify the cell density precisely, the cross sections of explanted grafts were stained with DAPI. The cell density in the grafts wall was determined as cell number/HPF (high power field) (×200). A: Representative DAPI staining of the cross-sections of the explanted grafts. B: Quantitative analysis of cell density. Ten high-magnification DAPI images per sample, five samples at 1month and four samples at 3 months per group were included to quantify the cell density.

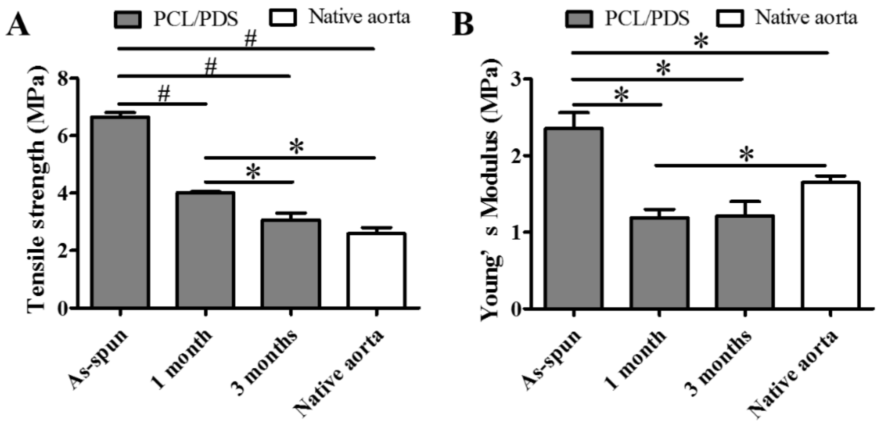

Fig. S9 The transverse mechanical properties of the explanted PCL/PDS vascular grafts (n = 3) and native aorta (n = 3). A: Tensile strength. B: Young's Modulus. $^{*}p < 0.05$, $^{\#}p < 0.001$.

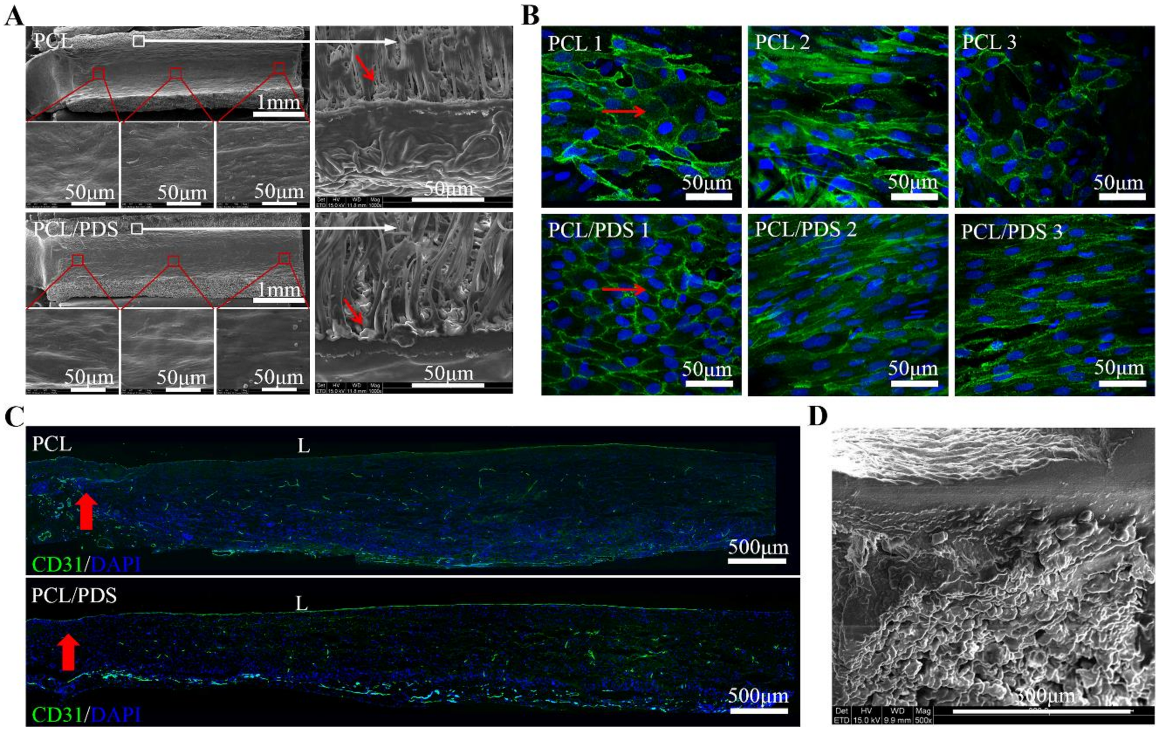

Fig. S10 Endothelialization formation of the expanded grafts after 3monthsimplantation. A: The lumen surface of explanted grafts was observed by SEM (red arrows: collage). B: The endothelial coverage of expanded grafts was observed by *En face* immunostaining using CD31 antibody at three different positions, including the near suturing site (1), the midportion (3) and the site between them (2) (Red arrows: blood flow direction). C: Endothelialization was analyzed by immunofluorescence staining of longitudinal sections of grafts using CD31 antibody (L: lumen; Red arrows: suture site). D: SEM image of native abdominal aorta vessel.

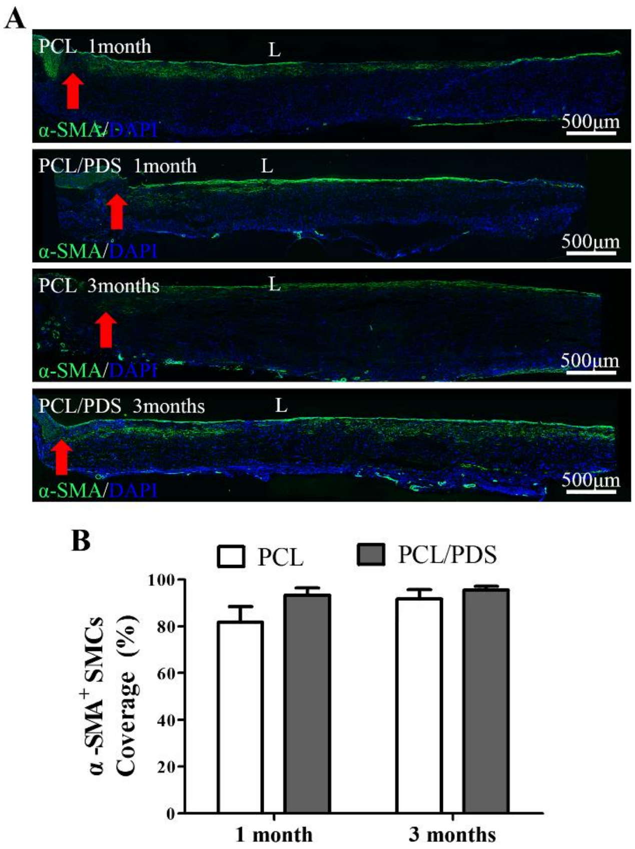

Fig. S11 Synthetic smooth muscle regeneration of explanted grafts at 1 and 3 months after implantation. A: Longitudinal sections were stained with anti-smooth muscle actin (α-SMA) antibody to identify the coverage of synthetic phenotype SMCs. B: The synthetic phenotype SMCs coverage rate of grafts was calculated based on α-SMA staining. L: lumen; Red arrows: suture site.

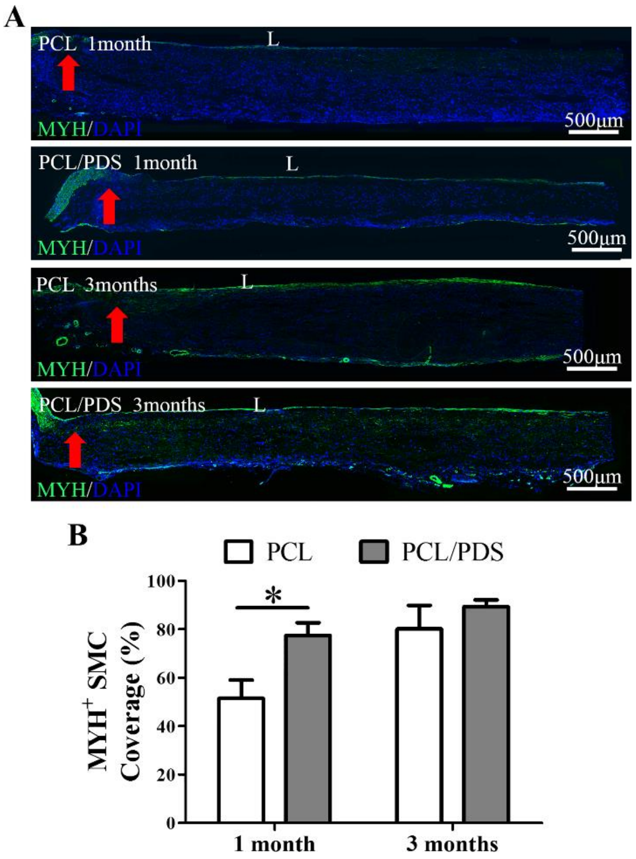

Fig. S12 Contractile smooth muscle regeneration of explanted grafts at 1 and 3 months after implantation. A: Longitudinal sections were stained with anti-smooth muscle myosin heavy chain I (MYH) antibody to identify the coverage of contractile phenotype SMCs. B: The contractile phenotype SMCs coverage rate of grafts was calculated based on MYH staining. $^{*}p < 0.05$. L: lumen; Red arrows: suture site.

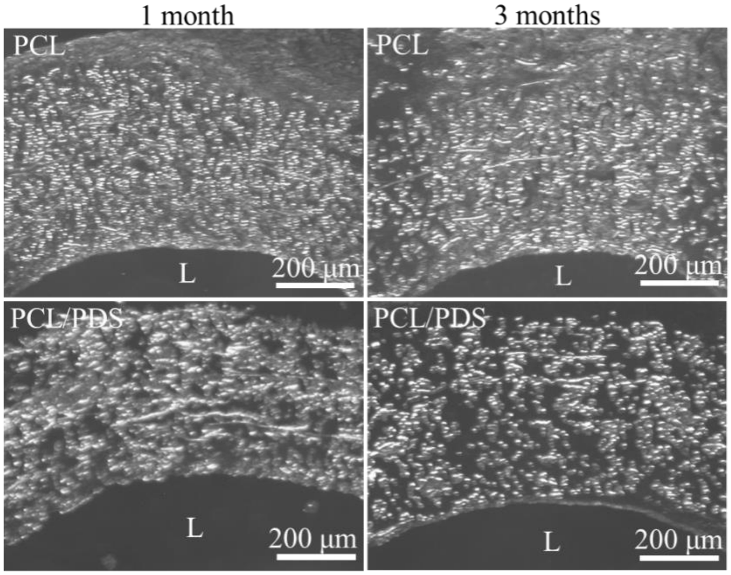

Fig. S13 The degradation of the electrospun PCL, PCL/PDS grafts at 1 and3 months after implantation. The change of the explanted graft wall was observed by stereomicroscope using HMC method. (L: lumen).

Table S1. The electrospinning parameters of different ratio of PCL/PDS grafts.

PCL:PDS (w/w)	Flow rate (ml/h)		Feasibility
	25% PCL	20% PDS	
2.50:1	8	4	No
1.67:1	8	6	Yes
1.00:1	8	10	No
0.60:1	8	16.67	No

Reference

1. Wang, S., Zhang, Y., Wang, H., Yin, G. & Dong, Z. Fabrication and properties of the electrospun polylactide/silk fibroin-gelatin composite tubular scaffold. *Biomacromolecules* 10, 2240-2244 (2009).
